# Validation of the Activity Preference Assessment: a tool for quantifying children’s implicit preferences for sedentary and physical activities

**DOI:** 10.1186/s12966-020-01014-6

**Published:** 2020-08-24

**Authors:** S. Nicole Fearnbach, Corby K. Martin, Steven B. Heymsfield, Amanda E. Staiano, Robert L. Newton, Alex C. Garn, Neil M. Johannsen, Daniel S. Hsia, Owen T. Carmichael, Sreekrishna Ramakrishnapillai, Kori B. Murray, John E. Blundell, Graham Finlayson

**Affiliations:** 1 Clinical Sciences Division, Pennington Biomedical Research Center, Baton Rouge, Louisiana USA; 2 Population and Public Health Sciences Division, Pennington Biomedical Research Center, Baton Rouge, Louisiana USA; 3 Louisiana State University, Baton Rouge, Louisiana USA; 4 University of Leeds, Leeds, England, UK

**Keywords:** Decision-making, Sedentary behavior, Obesity, Fitness, Pediatrics

## Abstract

**Background:**

High levels of sedentary behavior and low physical activity are associated with poor health, and the cognitive determinants of these behaviors in children and adolescents are not well understood. To address this gap, we developed a novel, non-verbal, computer-based assessment to quantify the degree to which youth prefer to be sedentary relative to physically active in their leisure time.

**Methods:**

The Activity Preference Assessment (APA) uses a forced-choice paradigm to understand implicit decision-making processes when presented with common sedentary and physical activities. The APA bias score ranges from − 100 to + 100, with positive scores indicating a relative preference for sedentary activities, and negative scores representing a preference for physical activities. In 60 children ages 8–17 years, we assessed the validity of this behavioral task against a free-choice play observation, accelerometry-measured activity, anthropometrics and body composition, and cardiorespiratory fitness. We explored neighborhood, family, and individual-level factors that may influence implicit activity preferences. Test-retest reliability was assessed over one week.

**Results:**

The majority of children (67%) preferred sedentary relative to physical activities. APA bias scores were positively associated with sedentary time during free-choice play. In girls, bias scores were negatively associated with average daily MVPA. APA bias scores were positively associated with body fat and negatively associated with cardiorespiratory fitness. These findings were independent of age, sex, and race/ethnicity. Neighborhood access to physical activity spaces, the number of people in the home, perceived physical self-competence (e.g., coordination, strength), and self-reported depressive symptoms were associated with activity preferences. The intra-class correlation for test-retest reliability was *r* = 0.59.

**Conclusions:**

The APA shows promise as a novel tool for quantifying children’s relative preference for sedentary versus physical activities. Implicit bias scores from the APA are clinically meaningful, as shown by significant associations with adiposity and cardiorespiratory fitness. Future longitudinal studies should examine the directionality of the association between preferences and health markers, and the degree to which implicit activity preferences are modifiable. Importantly, the task only takes an average of 10 min to complete, highlighting a potential role as an efficient screening tool for the propensity to be sedentary versus physically active.

**Trial registration:**

ClinicalTrials.gov NCT03624582.

## Introduction

High levels of sedentary behavior and low physical activity are independently associated with adverse metabolic health outcomes, including obesity and diabetes [1–4]. Results of a recent meta-analysis suggest that sedentary time is positively associated with risk for obesity and poor metabolic health during childhood, which contributes to the elevated risk for type 2 diabetes, cardiovascular disease, and all-cause mortality in adulthood [5]. Prolonged sedentary time is positively associated with adiposity and waist circumference in children [6]. Obesity-related behaviors tend to track throughout the lifespan, further exacerbating the risk for chronic conditions as we age [7]. It is important to identify the factors that contribute to children’s desire to be sedentary rather than physically active. Understanding the early cognitive determinants of these habits is necessary to promote improvements in long-term health [8, 9], especially in children with a propensity to be sedentary in their leisure time.

One of the major cognitive factors studied in regards to physical activity is motivation. There is a posited role for intrinsic motivation in the determination of autonomous activity behaviors [10], consistent with the Theory of Self-Determination [10, 11]. Individuals on the controlled motivation end of the spectrum are externally regulated and more likely to exercise for reward or punishment, whereas those on the autonomous motivation end of the spectrum are intrinsically motivated and more likely to exercise for enjoyment, pleasure, and fun. People with an intrinsic motivation report greater exercise intentions, exercise more frequently, and derive a greater sense of well-being from exercise [10]. The same may be true for sedentary behaviors, although studied less frequently. The Affective-Reflective Theory [12] states that experiences, feelings, and thoughts surrounding exercise influence the willingness to undergo the same physical strain again, suggesting that negative affective responses promote sedentariness. The Theory of Energetic Cost Minimization [12] assumes that biomechanically efficient behaviors, such as sedentary behaviors, have an inherent rewarding value, which may limit the effects of intervention programs. In a recent study [13], motivation to be sedentary was assessed by a relative reinforcing value (RRV) task using a progressive ratio schedule of mouse clicks on a computer to earn access to one or both of the alternatives, as a measure of willingness to work for the “reward.” Results indicated that motivation to be sedentary limited the effectiveness of an intervention to reduce sedentary behavior [13]. However, the RRV task utilized in this investigation only compared the participants’ single favorite sedentary activity to their single favorite physical activity. Therefore, this task is not able to evaluate the nuanced decision-making around leisure time overall. While time spent engaging in the target behavior (e.g., sedentary video game play) was reduced with the sedentary behavior intervention, physical activity time did not increase [13], suggesting that participants may have substituted another sedentary activity (e.g., watching TV, reading a book, etc.) in place of the target behavior. For this reason, it is important to understand the individual preferences towards engaging in sedentary behaviors as a whole, and the likelihood of choosing a sedentary activity over a physical activity.

The Activity Preference Assessment (APA) is a novel, computerized behavioral task designed to assess biases in decision-making across multiple leisure time activities. The APA is based on the psychometric properties and task design of a widely used and well-validated measure of explicit liking and implicit wanting for different types of food [14, 15], which has been shown to correlate with objectively measured food intake, self-reported eating behaviors, and markers of obesity [16–19]. We have applied this framework to assess similar cognitive constructs in reference to physical and sedentary activity preferences. High levels of sedentary behavior are associated with adverse health outcomes [5, 6], but the cognitive determinants of these habits are not well understood [20]. Questionnaires were previously developed to assess where people fall on the spectrum of exercise motivation described above [21–25], which primarily rely on explicit self-reported ratings of individuals’ reasons for exercising. While explicit reasoning is important, these measures don’t explore underlying cognitive processes. In addition, these methods may be susceptible to social desirability bias, depending on the context in which they are administered (e.g., enrollment in a weight loss trial). Implicit association tasks [26, 27] aim to overcome this problem by examining automatic evaluations of physical activities through associations with words like “pleasant” or “relaxing”. However, these tasks tend to examine activity types in isolation, and word association tasks of this type may not be developmentally appropriate for children of all ages. We are unaware of any existing tools that measure the predisposition to choose sedentary versus physical activities, and none that effectively quantify the implicit decision-making processes that may bias children towards these behaviors. The APA is designed to address this gap by quantifying the implicit bias for sedentary relative to physical activities, when children are given a choice for how to spend their leisure time.

This study enrolled 60 children to assess the convergent validity of the APA against leisure activity choices in a 30-min free-choice play period and seven days of accelerometry. We hypothesized that children with a relative preference for sedentary activities would spend more time sedentary and less time physically active. We also assessed criterion validity against measures of cardiorespiratory fitness and body composition, with the hypothesis that children with a relative preference for sedentary activities would have poorer fitness and higher levels of body fat. We explored additional demographic and psychosocial factors that we expected to influence the propensity to prefer sedentary versus physical activities. Finally, we assessed the test-retest reliability of APA outcomes one week apart. We hypothesized similar reliability to that reported for food-related implicit biases with an intra-class correlation of 0.60 [14]. Overall, this study aimed to establish the APA as a useful research tool to quantify children’s implicit preferences for sedentary and physical activities.

## Methods

### Participants

This validation study was an ancillary to a larger, ongoing cross-sectional study of children’s body shape, composition, and cardiometabolic health (Shape Up! Kids, NIH R01DK111698). We recruited a subsample of 60 of these children between the ages of 8–17 years, inclusive, to participate in the validation study. Participants were considered eligible for the study if they did not have any physical or medical conditions that prohibited them from being physically active, did not have any contraindications to exercise testing as defined by the American College of Sports Medicine (ACSM), and did not have any contraindications to body composition imaging techniques (e.g., metal in or on the body that could not be removed). Parents or legal guardians signed written informed consent for all procedures in this study, including parent-reported questionnaires and all measures completed by the child. Children 11 years and younger provided verbal assent and children 12 years and older provided written assent to participate. All study procedures were approved by the Institutional Review Board of Pennington Biomedical Research Center.

### Study design

Participants attended two study visits at the Center. For the main Shape Up! Kids study, participants completed a fasted clinic visit in the morning where anthropometrics, body shape, body composition, vital signs, and fasting blood assays were collected. At this same visit, children completed the APA (Time 1) and parents completed a questionnaire regarding family demographics and family medical history. Participants were given an accelerometer to wear for seven days between visits. They then returned for a second clinic visit, which took place 8–21 days after the initial visit, depending on the family’s availability. Participants returned the accelerometer, completed the APA (Time 2), were observed during a 30-min free-choice play period, filled out self-report questionnaires, and underwent a VO_2max_ cardiorespiratory fitness test on a cycle ergometer. A parent or guardian filled out a neighborhood and home environment survey.

### Measures

#### The Activity Preference Assessment (APA)

**Fig. 1:**
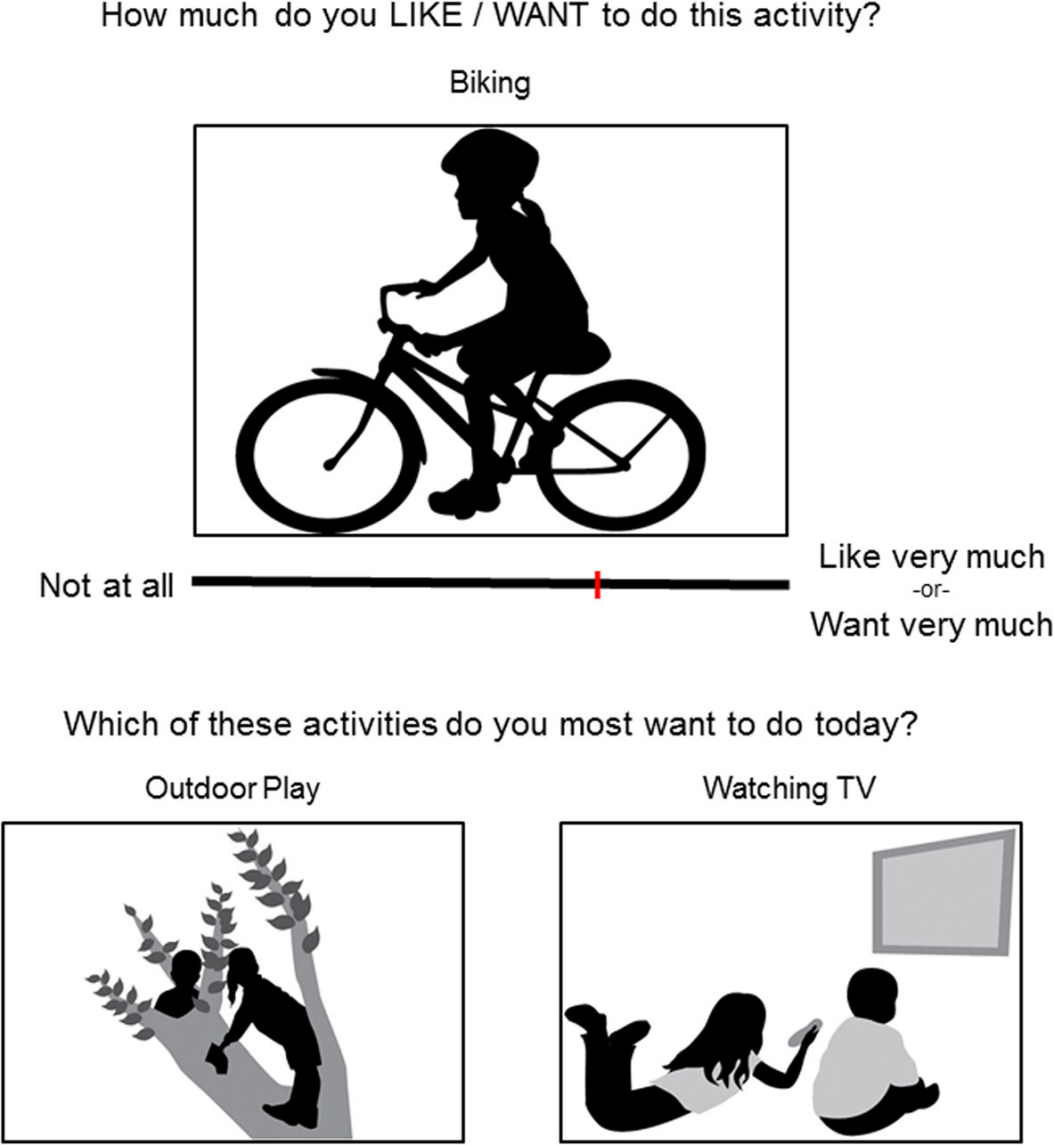
The Activity Preference Assessment task design. Top: Visual Analog Scales (VAS), Bottom: Forced-choice paradigm

The APA task consists of two parts (Fig. 1) and is administered on a desktop computer via E-Prime (Psychology Software Tools, Sharpsburg, PA, USA). The task takes approximately 10 min to complete. Each participant is first asked to rate how much they *like* to do and *want* to do a variety of common physical activities and sedentary activities (*Physical*: ball sports, biking, dancing, gymnastics/tumbling, outdoor play, running, swimming, walking; *Sedentary*: arts and crafts, board games, tablet, listening to music, reading, talking, watching TV, video games) using visual analog scales (VAS). Following completion of the VAS section, they complete a non-verbal forced-choice paradigm. Out of each pair of activity images (four sets of 30 pairs, with breaks between sets), they are asked to select as quickly as possible the activity they most want to do to assess non-verbal decision-making. Sixty-four of the 120 pairs are sedentary versus physical activities, with the remaining pairs falling within-category (28 sedentary, 28 physical activity). Every pair is unique and all possible comparisons are made. The two parts of the task are distinct and scores from each are not reliant on one another.

From the APA, we quantify a variety of outcomes. From the VAS, we quantify explicit liking and wanting ratings for individual sedentary activities and physical activities, and the average across each category, with a possible range of scores from 0 to 100. From the forced-choice section, we use an algorithm to calculate implicit bias scores (possible range − 100 to + 100) based on “wins” (which choice was selected) and reaction times in head-to-head comparisons of sedentary versus physical activities (64 total trials). The method for calculation of bias scores is below:

$$ {\displaystyle \begin{array}{l}{IW}_{SED}=\left[{WIN}_{SED}\ast \left({RT}_{ALL}/{RT}_{SED}\right)\right]-\left[{WIN}_{PA}\ast \left({RT}_{ALL}/{RT}_{PA}\right)\right]\\ {}{IW}_{PA}=\left[{WIN}_{PA}\ast \left({RT}_{ALL}/{RT}_{PA}\right)\right]-\left[{WIN}_{SED}\ast \left({RT}_{ALL}/{RT}_{SED}\right)\right]\\ {} BIAS\_ SCORE={IW}_{SED}-{IW}_{PA}\end{array}} $$

Wherein SED = Sedentary Activity and PA = Physical Activity; IW = Implicit Wanting for SED or PA, respectively; WIN = # of times SED or PA was selected, respectively; RT = Reaction Time for ALL trials of SED vs. PA, SED wins, or PA wins, respectively.

Positive (+) scores indicate a relative preference towards sedentary activities and negative (−) scores represent a relative preference towards physical activities. This implicit bias algorithm was originally developed for the Leeds Food Preference Questionnaire (LFPQ), which uses a similar paradigm as the APA [14]. Data is processed via an automated scoring procedure in Anaconda3 (Austin, TX, USA).

#### Free-choice play period

Participants were given 30 min to play freely by themselves with a variety of exercise equipment and sedentary games and activities from which to choose, which parallel those represented in the APA. Example physical activity options included a kid-friendly stationary bike and treadmill, a jump rope, a hula hoop, and active video games. Example sedentary activity options included reading books, a puzzle, an iPod touch, coloring supplies, and sedentary video games. A researcher instructed the participants on all of the available equipment and games and answered any questions prior to the start of the play period. They were told that they were allowed to play in this room for 30 min and use the time however they wish. There was a clock on the wall if they wanted to monitor their time. A single researcher observed through a two-way mirror from the adjoining room and recorded the participants’ self-selected activities in 15-s intervals. Inter-rater reliability was confirmed by having a second observer at 10 of the 60 visits and demonstrated 91.4% agreement. A group of assessors was trained in this method, adapted from the System for Observing Play and Leisure Activity in Youth [28], and previously used in a similar setting [29]. From this observation, we calculated the percent of the total time spent sedentary (i.e., not standing or moving) for analysis.

#### Accelerometry

Habitual physical activity and sedentary time were measured by a tri-axial accelerometer (ActiGraph GT3X+, ActiGraph, Pensacola, FL, USA). The participant was instructed to wear the accelerometer on an elasticized belt on the right hip, 24-h per day for at least 7-days (plus an initial familiarization day and the morning of the final day), including 2 weekend days. The minimal amount of accelerometer data that was considered acceptable was 4 days with at least 10 h of awake wear time per day (excluding the sleep period), including at least one weekend day. Upon returning the device, the research team verified the data for completeness using the ActiLife software (version 6.13.4; ActiGraph, Pensacola, FL, USA). The research team asked children to wear the accelerometer for additional days (to a maximum of 14 days) if the minimal data requirements were not met during the first wear period. Cut-points were assigned based on Evenson et al., with 0 to 25 counts per 15-s epoch (CPE) classified as sedentary, 26 to 573 CPE as light, 574 to 1002 CPE as moderate, and ≥ 1003 CPE as vigorous. The Evenson et al. cut-points were selected because they have been validated in this age range [30]. Total minutes and percent of time spent in each activity level (sedentary, moderate-to-vigorous physical activity [MVPA]) were extracted for analysis.

#### Cardiorespiratory fitness

Cardiorespiratory fitness (VO_2peak_) was determined with a graded cycle ergometer test with the use of standard open-circuit metabolic cart (Parvo Medic, TrueOne 2400, Sandy, UT, USA) until volitional fatigue. This protocol was designed to measure steady state responses to exercise at multiple submaximal stages leading up to a maximum workload. Prior to beginning the test, participants underwent a 5-min warm up on the cycle ergometer (Lode, Groningen, The Netherlands) to ensure comfortable positioning on the bike and to practice pedaling at the appropriate cadence (60 rotations per minute), which was maintained throughout the test. Two protocols were used, depending on the age and perceived fitness level of each participant at familiarization. Both protocols started with 3 min of unloaded pedaling at 0 W. In younger or less fit participants, the additional stages were 3 min long and increased in 20 W increments until at least 2 submaximal (loaded) steady state measurements were obtained, followed by 1 min stages increasing in 10 W increments until fatigue. In older or more fit participants, the workload for the 3 min stages increased in 35 W increments until at least 2 submaximal (loaded) steady state measurements were obtained, followed by 1 min stages increasing in 15 W increments until fatigue. All tests concluded with an active cool down phase and continued blood pressure and heart rate monitoring. The length of the test varied between participants, but was on average 12 min in duration. VO_2peak_ was achieved when at least two of the following criteria were met [31, 32]: 1) a plateau in VO_2_ (change < 2.1 mL/kg/min) with increasing workload, 2) a respiratory exchange ratio ≥ 1.0, 3) a heart rate ≥ 90% of the predicted maximum, or 4) a Rating of Perceived Exertion ≥19 [33]. Due to confounding effects of adiposity on fitness in children with obesity [34], VO_2peak_ was expressed relative to fat-free mass (mL/kg FFM/min) rather than total body weight.

#### Anthropometrics and body composition

Participants arrived to their initial Shape Up! Kids clinic visit after an overnight fast (10 h), confirmed by parental report. Height (cm), weight (kg), and body circumferences were measured by trained staff according to standard clinical procedures. These values were used to calculate BMI and age- and sex-specific BMI percentiles [35]. Adolescents with a BMI percentile ≥85 but < 95 were considered overweight, those with a BMI percentile ≥95 were considered to have obesity, and severe obesity defined as ≥120% of the 95th BMI percentile for age and sex. Total body fat and fat-free mass were measured with whole-body dual-energy X-ray absorptiometry (DXA) (Hologic Discovery System, Mississauga, ON, Canada). The participant was carefully positioned lying down on the table and asked to remain completely still as the detector passed over their body. Participants were scanned in duplicate. Percent body fat (%) was computed as total body fat (kg) divided by weight (kg) times 100. Fat mass index (kg/m^2^) was computed as total body fat (kg) divided by height (m) squared.

#### Questionnaires

Demographic and psychosocial factors may influence children’s engagement in sedentary time and physical activity. Therefore, we collected a Family Demographics and Family Medical History Survey, as well as a Neighborhood and Home Environment Survey from parents. We extracted children’s age, sex, and race/ethnicity, parents’ self-reported BMI, parents’ education, household income, the number of people living in the home, and children’s school type. We also examined parents’ reports of 1) access to neighborhood physical activity spaces, 2) neighborhood cohesion, 3) involvement in their children’s physical activity, 4) avoidant parenting behaviors, and 5) defensive parenting behaviors. Children completed the Physical Self Description Scale [36], Body Esteem Scale [37], and Mood and Feelings Survey [38] to assess potential influences of self-competence, body image, and depressive symptomatology on the inclination towards sedentary behaviors. The validity and reliability of each of these surveys have been previously reported in youth [36–38]. The Physical Self Description Scale Short Form includes 40 items self-rated by the child on a 6-point Likert scale (1 = False, 6 = True), with the following subscales: Health, Coordination, Activity, Body Fat, Sport, Global Physical, Appearance, Strength, Flexibility, Endurance, and Global Esteem. The Body Esteem Scale includes 23 items self-rated by the child on a 5-point Likert scale (1 = Never, 5 = Always), with the following subscales: Appearance, Weight, and Attribution. The Mood and Feelings Questionnaire Short Version includes 13 items self-rated by the child on a 3-point scale (0 = Not True, 1 = Sometimes, 2 = True), with the total score ranging from 0 to 26.

### Statistical approach

#### Power

Given that the APA is a novel method, we based our sample size calculations on previous studies of the LFPQ, which shares similar methods and scoring procedures. The minimum sample size to achieve a correlation of *r* ≥ 0.30 between LFPQ bias scores and eating behaviors was 30 participants [16]. We therefore enrolled a sample of 60 participants to achieve an even split of 30 boys and 30 girls, which allowed us to explore sex differences in the association between APA bias scores and activity behaviors or health markers.

#### Analysis strategies

Participant characteristics were generated by descriptive analysis (frequencies, means, standard deviations, ranges), and differences by sex and race/ethnicity were examined with independent samples t-tests. We used Pearson’s correlations to test the associations between Time 1 APA bias scores and the following variables: % of time spent sedentary in free-choice play, % and minutes/day of sedentary time and MVPA from accelerometry, VO_2peak_ (mL/kg FFM/min), BMI z-score, % body fat, and fat mass index. We also conducted partial correlation analysis controlling for age, sex, and race/ethnicity. Correlation analysis was used to test the associations between APA bias scores and questionnaire measures (demographic and psychosocial factors), with and without controlling for child BMI z-score. Test-retest reliability from Time 1 to Time 2 was assessed with intra-class correlation (two-way mixed, absolute agreement). Questionnaire data were collected and managed using Research Electronic Data Capture (REDCap) tools [39]. Data were analyzed in SPSS version 25 (IBM SPSS Statistics, New York, NY, USA). Results were considered significant at *p* < 0.05.

## Results

### Participant characteristics

Sixty participants (50% female) completed the study, with participant characteristics for the full sample and by sex described in Table 1. Notably, 28% of participants in this study were homeschooled at the time of enrollment.

**Table 1:** Participant characteristics and validation measures

	All (*n* = 60)	Boys (*n* = 30)	Girls (*n* = 30)
	n (%)	n (%)	n (%)
Age			
*8–12 years*	29 (48%)	16 (53%)	13 (43%)
*13–17 years*	31 (52%)	14 (47%)	17 (57%)
Race/Ethnicity			
*Non-Hispanic White*	25 (42%)	14 (47%)	11 (37%)
*Non-Hispanic Black*	22 (37%)	10 (33%)	12 (40%)
*Hispanic White*	8 (13%)	3 (10%)	5 (17%)
*Asian*	3 (5%)	1 (3%)	2 (7%)
*Multi-racial*	2 (3%)	2 (7%)	0 (0%)
Weight Status			
*Underweight*	3 (5%)	2 (7%)	1 (3%)
*Healthy Weight*	29 (48%)	17 (57%)	12 (40%)
*Overweight*	11 (18%)	3 (10%)	8 (27%)
*Obesity (not severe)*	12 (20%)	7 (23%)	5 (17%)
*Severe Obesity*	5 (8%)	1 (3%)	4 (13%)
	**Mean (SD)**	**Mean (SD)**	**Mean (SD)**
BMI Percentile	67.8 (31.4)	63.9 (31.8)	71.7 (31.0)
BMI Z-score	0.69 (1.24)	0.52 (1.26)	0.87 (1.22)
% Body Fat *	31.5 (9.0)	27.6 (9.3)	35.5 (6.9)
VO_2peak_ (mL/kg FFM/min)	40.5 (8.1)	42.5 (7.1)	38.8 (8.7)
% of Time Sedentary in Free-Choice Play Period	57 (36)	56 (35)	57 (37)
Daily Wear Time (min)	1074 (103)	1074 (111)	1075 (96)
Daily Sedentary Time (min) *	151 (49)	138 (41)	165 (53)
Daily MVPA (min) *	45 (17)	50 (19)	40 (13)

Note: Sample sizes vary for some measures due to criterion for data inclusion. *n* = 30 for VO_2peak_; *n* = 52 for Accelerometer Wear Time, Daily Sedentary Time, and Daily MVPA

^a^Significantly different between boys and girls at *p* < 0.05

**Fig. 2:**
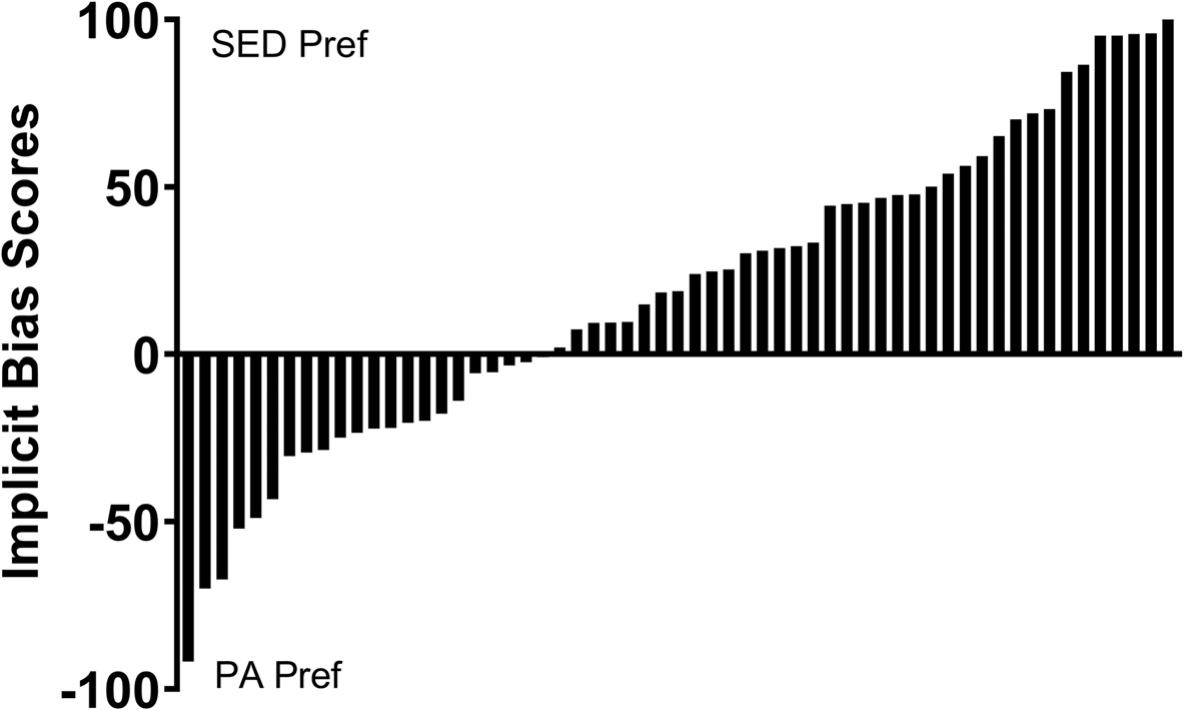
Waterfall plot of the distribution of individual Time 1 APA implicit bias scores across the cohort (n = 60). Positive scores indicate a relative preference for sedentary activities (SED Pref), while negative scores indicate a relative preference for physical activities (PA Pref). Two-thirds (67%) of participants preferred sedentary relative to physical activities

Time 1 APA bias scores revealed that 67% of participants had a preference for sedentary relative to physical activities (Fig. 2). The remaining descriptive data from the VAS and forced-choice sections of the APA are in Table 2. APA bias scores were not significantly associated with age (*p* = 0.23) and did not differ by sex (*p* = 0.24), but there were marginally higher scores (preference for sedentary relative to physical activities) among racial/ethnic minority children compared to white children (28.7 vs. 5.3; *t* = 1.97, *p* = 0.053). There were no differences in any variables (APA or validation measures) between homeschool and traditional schooling (all *p* > 0.25), nor between summer assessments and school-year assessments (all *p* > 0.13).

**Table 2:** Time 1 Activity Preference Assessment Outcomes

	*Mean*	*SD*	*Range*
**Visual Analog Scales (0–100)**			
Sedentary Activity Liking	70.8	14.2	26.4–99.6
Physical Activity Liking	62.0	17.1	16.6–93.0
Sedentary Activity Wanting	65.2	15.5	22.1–99.9
Physical Activity Wanting	54.7	19.4	4.6–90.8
**Forced-Choice Paradigm (−100–100)**			
Implicit Bias Score	18.8	46.2	−91.7 – 99.8

### Convergent validity

Descriptive results from the 30-min free-choice play period are described in Table 1. On average, children spent 57% (17 min) of their play time in sedentary activities. APA bias scores were positively associated with the % of time spent sedentary during the free-choice play period (*r* = 0.38, *p* = 0.004, *n* = 60), independent of age, sex, and race/ethnicity. In other words, children with a preference for sedentary relative to physical activities spent more time sedentary in a free play scenario. Fifty-two children had sufficient accelerometry data for analysis, with descriptive accelerometry results described in Table 1. There were no significant associations between APA bias scores and habitual activity (average daily sedentary time or MVPA) assessed by accelerometry, after controlling for age, sex, and race/ethnicity (all *p* > 0.62). In subgroup analysis, the only significant association was between APA bias scores and average minutes per day of MVPA in girls (*r* = − 0.41, *p* = 0.04; *n* = 25). In other words, girls with a preference for physical relative to sedentary activities spent more time engaging in daily MVPA.

### Criterion validity

**Fig. 3:**
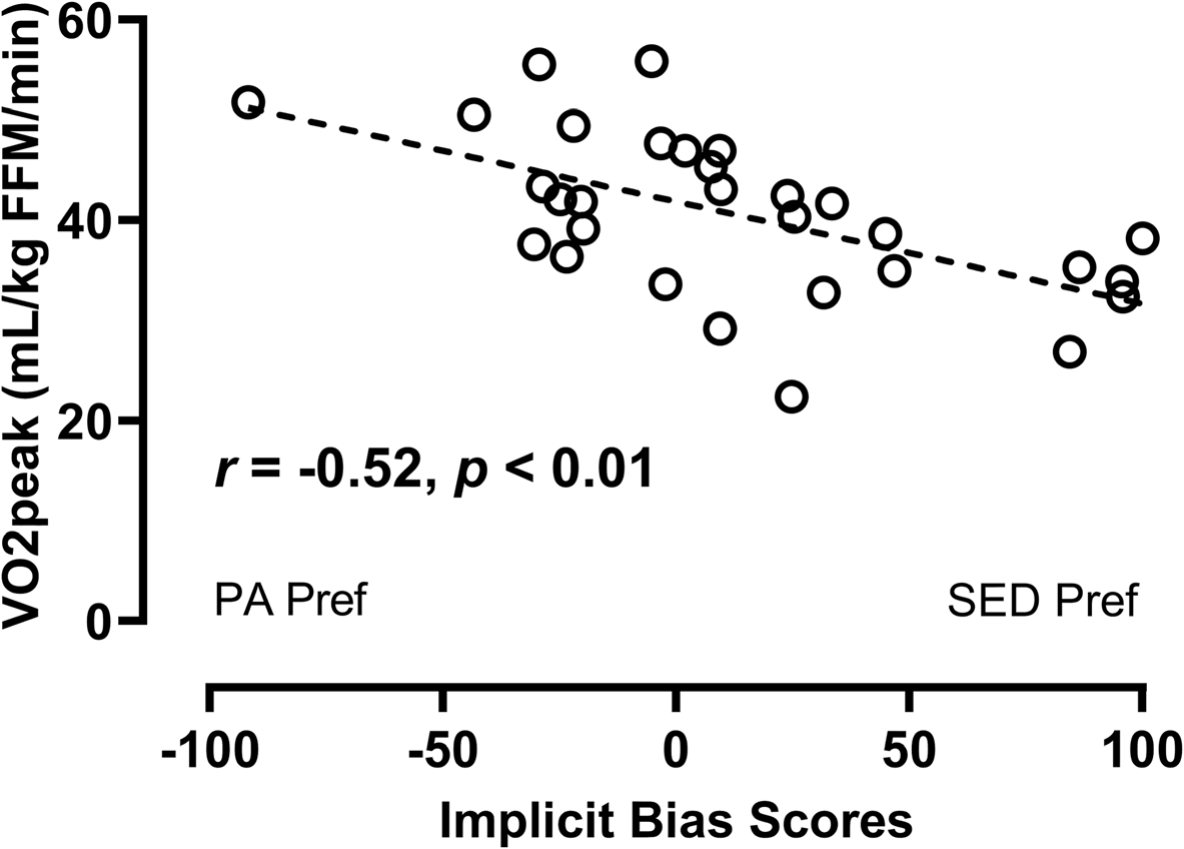
Correlation between APA implicit bias scores and cardiorespiratory fitness (VO_2peak_; mL/kg FFM/min), controlling for age, sex, and race/ethnicity (*r* = − 0.52, *p* = 0.005; n = 30). Positive scores indicate a relative preference for sedentary activities (SED Pref), while negative scores indicate a relative preference for physical activities (PA Pref)

**Fig. 4:**
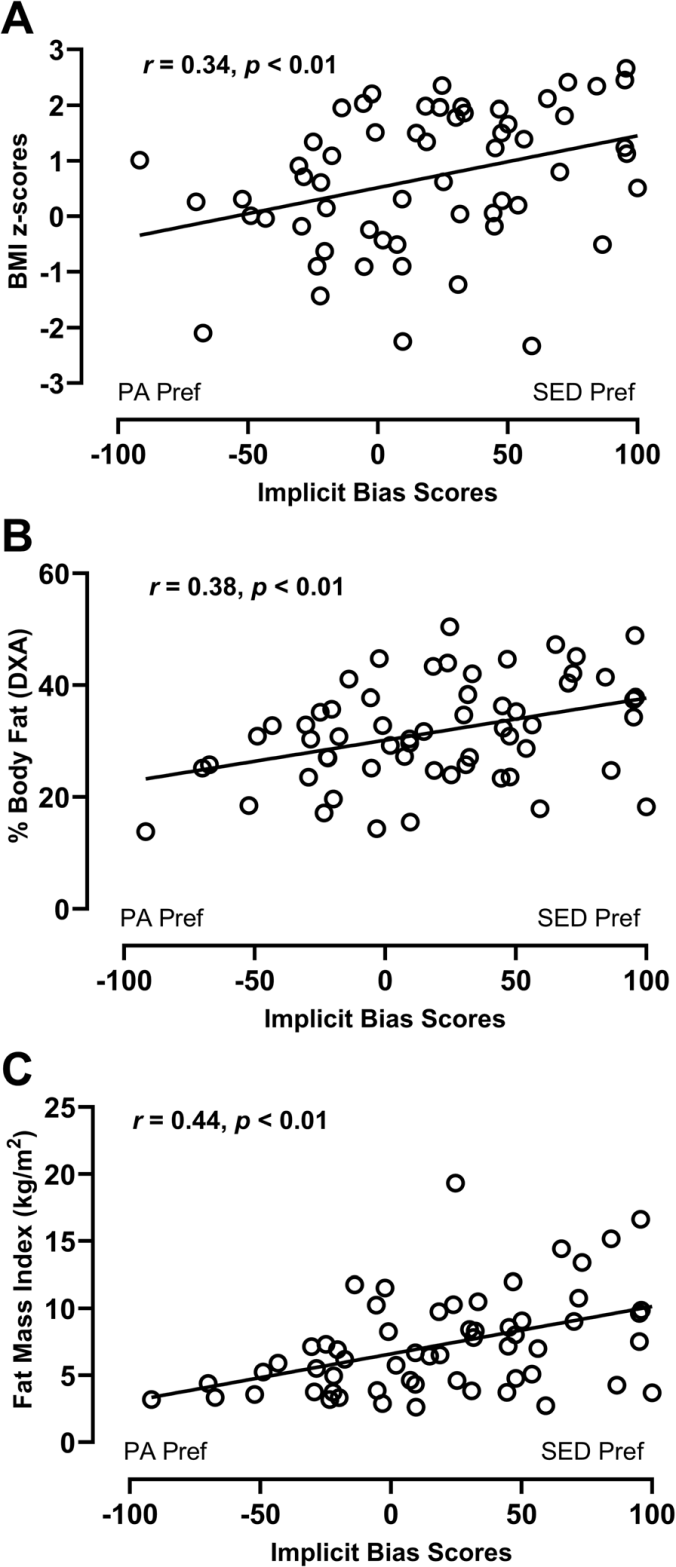
Correlations between APA implicit bias scores and indices of adiposity, controlling for age, sex, and race/ethnicity: **a** BMI z-score, **b** percent body fat, **c** fat mass index (kg/m^2^) (all *p* < 0.01; *n* = 60). Positive scores indicate a relative preference for sedentary activities (SED Pref), while negative scores indicate a relative preference for physical activities (PA Pref)

Thirty children met criteria for VO_2peak_, with descriptive cardiorespiratory fitness results described in Table 1. Children who met criteria for VO_2peak_ did not differ from those who did not, on any demographic characteristics or on APA bias scores (all *p* > 0.20, data not shown). APA bias scores were negatively associated with VO_2peak_ (mL/kg FFM/min) (*r* = − 0.52, *p* = 0.005; Fig. 3), independent of age, sex, and race/ethnicity. Children with a preference for sedentary relative to physical activities had lower cardiorespiratory fitness. APA bias scores were positively associated with all indices of weight status or adiposity (BMI z-score: *r* = 0.35, *p* = 0.008; % body fat: *r* = 0.43, *p* = 0.001; fat mass index [kg/m^2^]: *r* = 0.44, *p* = 0.001; Fig. 4), controlling for age, sex, and race/ethnicity. In other words, children with a preference for sedentary relative to physical activities had higher adiposity.

### Questionnaires

Children’s APA bias scores were not significantly associated with reported family income or parent education (all *p* > 0.14); however, they were negatively associated with the number of people in the home (*r* = − 0.35, *p* = 0.006). Children with more people in the household had a stronger preference for physical relative to sedentary activities. Children’s APA bias scores were also positively associated with their mother’s reported BMI (*r* = 0.39, *p* = 0.003), but not after controlling for child BMI z-score (*r* = 0.36, *p* = 0.06). There was no association with father’s reported BMI (*p* = 0.24). Children’s APA bias scores were negatively associated with parent-reported access to physical activity spaces in the neighborhood and home environment (*r* = − 0.32, *p* = 0.01), such that children with more access to physical activity spaces had a stronger preference for physical relative to sedentary activities. However, APA bias scores were not associated with parent-reported neighborhood cohesion, involvement in their child’s activity, avoidant parenting behaviors, or defensive parenting behaviors (all *p* > 0.14).

In regards to within-person factors, we found that APA bias scores were associated with several subscales from the Physical Self Description Questionnaire Table 3. Children who perceived themselves as more coordinated, stronger, more flexible, and having better endurance had a stronger preference for physical relative to sedentary activities (all *p* < 0.01). Conversely, children who perceived themselves as having higher body fat had a stronger preference for sedentary relative to physical activities (*p* < 0.01). There were no significant associations with subscales from the Body Esteem Scale, but there were trends for perceived appearance (*r* = − 0.24, *p* = 0.07) and weight (*r* = − 0.25, *p* = 0.06). Total scores from the Mood and Feelings Questionnaire were positively associated with APA bias scores (*r* = 0.32, *p* = 0.02), independent of child BMI z-score. Children who self-reported more depressive symptoms had a stronger preference for sedentary relative to physical activities, regardless of their weight.

**Table 3:** Pearson’s correlations between APA bias score and Physical Self Description Questionnaire subscales

	*r*	*p*
Health	0.22	0.09
Coordination **	−0.39	0.002
Activity	−0.06	0.63
Body Fat **	0.44	0.001
Sport	0.11	0.40
Global Physical	−0.20	0.13
Appearance	−0.21	0.11
Strength **	−0.39	0.002
Flexibility **	−0.40	0.002
Endurance **	−0.34	0.002
Global Esteem	0.14	0.29

Note: * *p* < 0.05, ** *p* < 0.01

### Test-retest reliability

Reliability for VAS outcomes was low. Intra-class correlations for each scale type were as follows: Sedentary Activity Explicit Liking, 0.32; Physical Activity Explicit Liking, 0.32; Sedentary Activity Explicit Wanting, 0.16; Physical Activity Explicit Wanting, 0.28. From the forced-choice paradigm, the intra-class correlation coefficient for APA bias scores was 0.59.

## Discussion

The purpose of this study was to assess the validity and reliability of scores from a novel, computerized behavioral task, the Activity Preference Assessment, designed to quantify non-verbal, implicit preferences for leisure time activities. The APA bias scores represent the degree to which a child implicitly prefers sedentary relative to physical activities, based on an algorithm that integrates choices and reaction times from a forced-choice paradigm. We hypothesized that APA bias scores would be positively associated with children’s time spent sedentary, as well as markers of weight status and adiposity. We also hypothesized that scores would be negatively associated with cardiorespiratory fitness. Our hypotheses were nearly all supported by the results from this study of 60 children varying in weight status and sociodemographic backgrounds. The only exception was a lack of association between APA bias scores and accelerometry-measured activity in boys. Overall, the convergent and criterion validity results from this study suggest that the APA is a tool that produces valid scores for assessing the underlying propensity to be sedentary versus physically active. Associations with adiposity and fitness were in similar directions to previous epidemiological reports of objectively-measured activity [3, 5, 6] and enhance the clinical meaningfulness of the APA bias scores in pediatric health research.

We found that children who preferred sedentary activities had higher levels of adiposity compared to children with a preference in the direction of physical activity. This might suggest that children with a relative preference for physical activity are “protected” against obesity. However, given the cross-sectional nature of the study, we cannot rule out that this relationship may be reciprocal. For example, children with obesity may have a stronger preference for sedentary activities due to physical or social limitations that often come with excess weight [40–43]. We propose a similar reciprocal relationship likely exists between cardiorespiratory fitness and APA bias scores, such that less fit children may be less inclined towards physical activity, and this in turn exacerbates lower fitness levels. Future longitudinal studies should clarify the directionality of these associations.

We also found APA bias scores to have very similar reliability to scores from the forced-choice paradigm in the LFPQ, which has a reported intra-class correlation of 0.6 and shares similar task design properties to the APA [14]. Given that the two assessments were separated by one week, it is possible that children’s preferences may have been influenced by personal experiences within that time frame. For this reason, the reliability could be tested further with repeated assessments over a shorter window (e.g., within the same day). In addition, future studies could help clarify whether activity preferences are a trait by examining the stability of APA bias scores over longer periods of time (e.g., six months to one year). However, unlike the constructs of food liking and food wanting in the ingestive behavior field, our liking and wanting VAS demonstrated low reliability. Outcomes from the VAS were also not associated with any of our validation measures (data not shown). At this point, we cannot recommend the use of VAS for understanding children’s activity preferences or their health risk, but future studies may clarify this discrepancy. Rather, it appears that non-verbal implicit preference and decision-making, as captured by the APA bias scores from the forced-choice paradigm, is a stronger determinant of behavior.

In this study, 67% of children showed a preference for sedentary relative to physical activities. Importantly, we investigated key demographic and psychosocial factors that we would expect to influence the desire to be sedentary versus physically active. In contrast to previous epidemiological studies showing that sedentary time increases in adolescence [44, 45], we did not see an association between activity preferences and age in our cohort. There was not a significant difference between boys’ and girls’ average APA bias scores, despite previous evidence suggesting that boys tend to be more physically active, particularly in the adolescent years [46]. Our data suggest that average APA bias scores may be higher among racial/ethnic minority compared to white children, although this trend did not reach statistical significance (*p* = 0.053). Larger studies focused on this particular question could clarify racial/ethnic differences further. We found that children with more people living in their home and with greater access to physical activity spaces in their home and neighborhood environment had a stronger preference for physical relative to sedentary activities. This highlights a potential role for modelling and the built environment in the underlying implicit preferences for leisure time activities. Similar external influences have been shown to affect food preferences and intake [47], drawing additional connections between the ingestive behavior and physical activity fields [48]. It is not surprising that energy balance related behaviors, as a whole, may be influenced through similar pathways [49, 50].

In regards to within-person factors, we anticipated that several cognitive constructs would influence the relative preference for sedentary versus physical activities. These included psychosocial measures of self-competence, body esteem, and depressive symptoms. We found that children who perceived themselves as more physically competent (better coordination, strength, endurance, and flexibility) had a stronger preference for physical relative to sedentary activities. This suggests that improving self-competence could influence a child’s underlying preference for physical activity and therefore promote engagement, but additional interventional studies are needed to investigate this approach. In addition, children who perceived themselves as having higher body fat and those who reported more depressive symptoms had a stronger preference for sedentary relative to physical activities. These findings are in line with previous studies of objectively measured activity [51, 52]. Importantly, the effects of depressive symptoms on activity preferences were independent of weight, despite many previous reports of associations between depression and obesity [53]. The findings from this study highlight the need to acknowledge individual differences and examine implicit activity preferences in context.

Some limitations should be noted. First, the APA is designed specifically to assess leisure time physical and sedentary activities and does not take into account activities associated with school or work. For this reason, we may have been limited in our ability to detect associations between APA bias scores and accelerometry-measured activity, a large proportion of which includes structured time. Future studies could examine factors that influence children’s autonomy over their activities in free-living settings, to potentially explain associations (or lack thereof) with accelerometer-measured activity levels. Similarly, accelerometer wear protocols could be adapted to specifically target and capture leisure time. For example, the current study only required one weekend day of valid data. The inclusion of the free-choice play period observation provided important additional insight into children’s leisure time habits. The activities in the free-choice play period were selected to mimic those in the APA as closely as possible, but considering the constraints of a clinical setting (i.e., only those that could fit within a single room). Given the significant associations between APA outcomes and actual time spent in sedentary activities during free-choice play, the findings have practical implications for classifying, predicting and potentially improving children’s leisure time activity patterns. Similar methodologies in other health behaviors, such as food preferences, have been used to evaluate treatments for obesity [54] and set dietary recommendations for military operations [55]. Research suggests leisure time activity preferences have similar efficacy for predicting real-life activity behaviors [56] as food preferences do for predicting actual food choice and intake. Therefore, future studies should extend this present work to children’s routine play settings like the home, a gymnasium, or outdoor environments. Further, the preference for sedentary activities and the association with engagement in such activities provides a cognitive construct that can be modified in an intervention, though future research is needed to examine if modifying this construct results in robust changes in leisure time activity. Finally, it is unclear if the state of the individual (e.g., tired, happy, etc.), time of day, or other acute factors affect APA bias scores. Strengths of this study include the diversity of the cohort in regards to demographics and weight status, allowing for better generalizability of the current findings. We also included gold standard, clinical measures of body composition and cardiorespiratory fitness. In addition, we took a rigorous, multi-level approach to assessing additional internal and external factors that could influence our novel measure of implicit activity preferences.

In conclusion, the Activity Preference Assessment shows potential as a tool for quantifying children’s underlying implicit preferences for leisure time activities, and demonstrated clinically meaningful associations with health status markers. More studies are now required to gain even more confidence in the status of the task as a reliable tool. Given the cross-sectional nature of the current study, future investigations should determine whether children with an implicit preference for sedentary activities are at increased risk for developing obesity and comorbid conditions over time. This 10-min task could be used as a screening tool in future research or clinical settings to identify children with a greater propensity to be sedentary, who may need more support for behavior change. Interventions could investigate the degree to which activity preferences are modifiable via strategies such as repeated exposure, which have been successful in other health domains (e.g., food preferences). Overall, the APA shows promise as a novel tool for pediatric obesity research.

## Data Availability

The dataset from the current analysis is not publicly available given that it is part of a larger, ongoing, clinical study, but is available from the corresponding author upon reasonable request.

## Abbreviations

APA: Activity Preference Assessment
BMI: Body mass index
CPE: Counts per epoch
DXA: Dual-energy x-ray absorptiometry
FFM: Fat-free mass
LFPQ: Leeds Food Preference Questionnare
MVPA: Moderate-to-vigorous physical activity
PA: Physical activity
Pref: Preference
RRV: Relative Reinforcing Value
SED: Sedentary activity
VAS: Visual Analog Scale
VO_2max_: Maximal oxygen uptake/aerobic capacity
VO_2peak_: Peak oxygen uptake/aerobic capacity
